# Reuse of cardiac organs in transplantation: an ethical analysis

**DOI:** 10.1186/s12910-018-0316-z

**Published:** 2018-08-17

**Authors:** Eisuke Nakazawa, Shoichi Maeda, Keiichiro Yamamoto, Aru Akabayashi, Yuzaburo Uetake, Margie H. Shaw, Richard A. Demme, Akira Akabayashi

**Affiliations:** 1 Department of Biomedical Ethics, Faculty of Medicine, The University of Tokyo, 7-3-1 Hongo, Bunkyo-ku, Tokyo, 113-0033 Japan; 2 Course for Health Care Management, Graduate School of Health Management/Department of Health Policy and Management, School of Medicine, Keio University, Kanagawa, Fujisawa, 252-0883 Japan; 3 Division of Medical Humanities and Bioethics, University of Rochester Medical Center, Rochester, NY USA; 4 Division of Medical Ethics, New York University School of Medicine, New York, NY USA

**Keywords:** Retransplantation, Reuse organ transplantation, Heart transplantation, Legal aspect, Ethical aspect, Property right, United States, Japan

## Abstract

**Background:**

This paper examines the ethical aspects of organ transplant surgery in which a donor heart is transplanted from a first recipient, following determination of death by neurologic criteria, to a second recipient. Retransplantation in this sense differs from that in which one recipient undergoes repeat heart transplantation of a newly donated organ, and is thus referred to here as “reuse cardiac organ transplantation.”

**Methods:**

Medical, legal, and ethical analysis, with a main focus on ethical analysis.

**Results:**

From the medical perspective, it is critical to ensure the quality and safety of reused organs, but we lack sufficient empirical data pertaining to medical risk. From the legal perspective, a comparative examination of laws in the United States and Japan affirms no illegality, but legal scholars disagree on the appropriate analysis of the issues, including whether or not property rights apply to transplanted organs. Ethical arguments supporting the reuse of organs include the analogous nature of donation to gifts, the value of donations as inheritance property, and the public property theory as it pertains to organs. Meanwhile, ethical arguments such as those that address organ recycling and identity issues challenge organ reuse.

**Conclusion:**

We conclude that organ reuse is not only ethically permissible, but even ethically desirable. Furthermore, we suggest changes to be implemented in the informed consent process prior to organ transplantation. The organ transplant community worldwide should engage in wider and deeper discussions, in hopes that such efforts will lead to the timely preparation of guidelines to implement reuse cardiac organ transplantation as well as reuse transplantation of other organs such as kidney and liver.

## Background

The term “retransplantation” refers to a repeat transplantation within a particular recipient. The recent literature includes a successful case of fourth-time cardiac retransplantation [1], eliciting ethical discussions about resource allocation and priorities among recipients, including those awaiting transplant [2, 3].

This paper addresses issues arising from an entirely different type of retransplantation. That is, transplantation of a donated heart from a first recipient (FR) to a second recipient (SR), when the FR becomes brain-dead at some point after receiving the heart from the first donor (FD). In this case, the FR becomes the second donor (SD), and is thus referred to as FR/SD. This type of retransplantation is referred to here as “reuse cardiac organ transplantation (RCOT)” as it involves the reuse of an already transplanted organ. A review of the current literature yields seven cases of RCOT [4–6], as well as other cases of reuse organ transplantation of the kidney [7–10] and the liver [11–13]. Graetz et al. discuss the ethical issues around consent from the deceased donor’s family for the reuse of a previously transplanted kidney [14]. While these issues overlap somewhat with those pertaining to reuse transplantation of organs such as the kidney or liver, the present study focuses on cardiac organ transplantation. We believe that an ethical analysis of cardiac organ transplantation should create the general framework for all other models of reuse organ transplantation, specifically because cardiac transplantation *is impossible for living donors.* The question is: what is the moral status of an organ transplanted into a recipient’s body? This paper presents a hypothetical case and discusses the medical, legal, and ethical issues therein.

## Methods

Medical, legal, and ethical analysis, with a main focus on ethical analysis.

### Case

Patient A, a thirty year-old male patient diagnosed with severe dilated cardiomyopathy, received a heart from a brain-dead donor in Japan. After transplantation, he followed an uneventful postoperative course for 6 months.

Subsequently, Patient A was involved in a traffic accident. He suffered severe injuries, including trauma to his head, and was transported to an emergency hospital. Upon arrival, physicians determined that Patient A met clinical criteria for brain death. From his personal belongings, the medical team retrieved a signed donor card that expressed his intention to donate all organs in case of brain-death. The card was also signed by his family. Given the documented consent by his family toward his intent for organ donation, the clinical team considered the indication of his intention valid and contacted the Japan Organ Transplant Network (JOTN) [15].

### Question

Are there any legal and ethical issues that may arise when SD’s heart, transplanted from FD, is re-transplanted into the body of another individual?

## Results

### Medical aspects

Currently, there is insufficient evidence to perform a risk-benefit assessment of RCOT. The medical team must evaluate the impact of the initial heart transplantation on the transplanted heart. Meanwhile, four additional risk factors are involved in RCOT. First, there is a risk of damaging the transplanted heart during organ retrieval from FR/SD, due to increased levels of difficulty associated with pericardial adhesions following the initial surgery. Second, the development of post-transplant coronary artery vasculopathy may pose issues. The longer the time period after the initial transplant, the higher the risk for SR, as the heart deteriorates over time. Seven of the cases reported thus far involved retransplantation within 16 days [4]. Third, the two separate ischemic periods that the heart undergoes may damage the right ventricular function. Finally, if either the heart or vessels to be retransplanted contain components from the previous donors, there may be increased immunological risk [16].

## Legal aspects

### Organ transplantation laws in the United States (US) and Japan

Legal requirements for organ retrieval from the body of a brain-dead individual for transplantation purposes are similar between the two countries [15, 17]; both the US and Japan require an indication of the donor’s intention. However, in Japan, the family’s approval is also required, so even if the intention to donate organs is expressed by the individual, their organs cannot be used for transplantation if the family does not also give consent. On the contrary, in the US, organ procurement is legally permissible in the presence of the donor’s intention, even over the family’s objection. While family members generally comply with the donor’s intention, there are cases in which family members object to donation in the US. Under such circumstances, while organ procurement is legally permissible, institutions have historically deferred to family wishes (no published data). Recently, however, institutions have increasingly honored donor’s expressed wishes over the objection of the family (no published data). In both countries, an opt-in system is in place for cases in which there is no expressed intention indicated. In summary, the individual’s intention is the most important requirement in the US, whereas in Japan, the approval of the family is also necessary.

### Organs for transplantation and their property rights

US courts have historically recognized the interests of family members in the body of the deceased person for the purposes of burial or cremation; however, courts have not recognized property rights in human body parts [18]. American legal scholars have argued for property rights in human biological materials to “promote donor autonomy” and to address the perceived injustice of the existing legal framework [19–22]. Opponents argue that property rights in human biological materials undermine the progress of biotechnology as well as the prohibition of organ sales under the National Organ Transplant Act and The Uniform Anatomical Gift Act [17, 23]. In a case involving a cell line developed from human tissue, the Supreme Court of California concluded that individuals do not have property interests in human tissue removed from their body [24].

In Japan, there was a case in which a university preserved autopsy organs as microscopic specimens, and the family of the deceased asked the university to hand over the specimens for the purpose of burial and religious services. In this case, a district court granted the family’s request to return the deceased’s body specimens based on the principle of ownership of the remains by the family [25]. Accordingly, discussions could potentially arise with respect to transplant organs as well, in relation to property rights in the future.

While mainstream opinions among lawyers in both countries do not acknowledge property rights in organs used for transplantation, Japan still requires further discussion on whether property rights in transplant organs should be recognized or not.

### Views on the legality of procuration of the transplanted organ from FR/SD

In the US, there is no legal issue associated with organ donation from FR/SD to SR. Similarly, in Japan, there is no legal issue with removing the transplanted organ from FR/SD and transplanting it to SR, even though the organ originates from FD. As such, in both the US and Japan, RCOT is legally permissible.

## Ethical aspects

### A working premise

Let us propose a working premise, based on which further ethical discussions will be carried out. Our working premise is as follows: RCOT is either ethically permissible or impermissible.

We first focus on the scope of consent for the two donors (i.e., FD and FR/SD). Should RCOT require consent from FD, as well as from FR/SD? If so, in cases where the contents of consent from each donor do not conform to one another, which donor’s intention will be given higher priority? We organized each intention of FD and FR/SD regarding RCOT into the following four patterns:

**Table:** 

	FD	FR/SD
Pattern 1	Agree	Agree
Pattern 2	Agree	Disagree
Pattern 3	Disagree	Agree
Pattern 4	Disagree	Disagree

For the sake of argument, in each Pattern, “Agree” means that FD or FR/SD gave their consent or expressed their wish for RCOT, whereas “Disagree” indicates the opposite. Under these conditions, is RCOT ethically permissible or impermissible?

### Argument 1 – RCOT is ethically permissible

In the case of Pattern 1, RCOT is ethically permissible from the perspective of donor consent.

Next, let us consider Pattern 3, that is, the case in which FR/SD wishes for RCOT, overriding the expressed wish of FD not to have the organ retransplanted.

In addressing this problem, a philosophical argument about the aforementioned “property rights,” along with a natural right theory or a philosophical/ethical theory based on self-ownership, might help establish a foothold. According to John Locke, “every Man has a *Property* in his own *Person.* This nobody has a right to, but himself. [26]” In other words, with regard to handling or disposing of one’s own body, the right to decide resides with that individual alone, not anyone else. In this context, our organs are our own possessions, and we have the freedom to donate them. Based on this concept of self-ownership, organ transplantation can be considered a transfer of one’s own property rights. Consider RCOT on the premise of self-ownership. In terms of the relations among the three—FD, FR/SD, and SR, currently the “ownership” of the heart at the time of reuse is no longer with FD, as it has been transferred from FD to FR/SD. At this point, FR/SD is able to donate the transplanted heart, irrespective of FD’s intention, as the owner of the heart. Therefore, in light of the philosophical/ethical self-ownership theory, the intention of FR/SD will be given priority.

Now, going back to Pattern 2 (i.e., FR/SD does not wish for RCOT despite the prior consent of FD; in this scenario, FR/SD is overriding the consent of FD), the idea that self-ownership of the organ has been transferred to FR/SD (as discussed in the case of Pattern 3) makes it ethically impermissible to carry out RCOT. Yet, is it possible to establish an ethical argument which can invalidate the aforementioned discussion based on “self-ownership”? In other words, what kind of ethical argument can be made, which allows the consent of FD to be prioritized over that of FR/SD and consequently makes RCOT ethically permissible?

Here we use the gift concept and heritage concept as candidates for such ethical arguments. The former is a concept relating to the human connection between a donor and recipient. When FD’s heart is transplanted into FR/SD, the transplanted heart can be regarded as a gift from FD to FR/SD. ‘Gift giving’ occurs (and is completed) when there is a sender and a receiver, and is usually accompanied by appreciation on the side of the receiver. As a token of appreciation, the receiver might consider giving that very gift they received to someone else as “a favor for favor,” when put in an appropriate position. In the case of heart transplantation, this ‘gratitude for FD’s favor’ cannot be expressed towards FD, who is already dead. Therefore, FR/SD’s gratitude could be directed towards SR, a third party, in the form of RCOT. Of course, we would not go so far as to say that FR/SD is always obligated to show gratitude in such a manner. However, if FR/SD denies consent for RCOT (to SR), this in it of itself might inevitably be considered a lack of gratitude toward FD. Such absence of gratitude toward FD can be ethically justified with self-ownership rights, which have been classified into a kind of perfect duty with legal rights, and in that sense, is similar to legal arguments. However, the gift concept described here goes beyond the call of duty, and embodies the concept of virtue ethics; namely, that of supererogation. Therefore, while FR/SD is neither going against the law nor breaching their duty by denying consent for RCOT, such behavior is likely to be considered as simply lacking in virtue.

Another direction to take is to regard organs as an entity that is closer to “heritage,” among all sorts of public property. In fact, with respect to a certain kind of heritage, we find it unethical to dispose of or modify them based solely on the values of those currently living. For instance, some think it is unforgivable to handle Michelangelo’s sculptures, or Rembrandt’s paintings, in ways that go against the artists’ will [27, 28], no matter who owns them. Similarly, another way of thinking is that the reused and transplanted heart of FR/SD is inherited by SR with the history that it was once the heart of FD (i.e., an inheritance), and that the intention of FD as the original donor should be respected, to the extent possible, at the point of performing RCOT. Based on these ideas, RCOT is considered ethically justifiable in the case of Pattern 2 as well.

Finally, in the case of Pattern 4 (i.e., neither FD nor FR/SD gives consent for RCOT), stating that RCOT is ethically permissible is equivalent to saying that donors’ intention is irrelevant for retransplantation. This claim may seem intuitively incorrect. However, it is possible to justify RCOT even in the case of Pattern 4, according to the nature of organs as public property. For instance, a policy could be adopted to allow donated hearts to be handled as a sort of public property (“public property theory”), thereby making them subject to redistribution [29]. In other words, ownership rights are abandoned when FD (i.e., donor) gives consent for organ donation, and FD’s organ is treated as a common resource from that point on (in a more limited sense, a resource for all recipients who are in need of that organ). This more radical position requires FD’s consent to be interpreted as “comprehensive” (i.e., to have their organs transplanted into multiple unspecified recipients). The public property theory grants FD, who is the original donor, no power to make decisions about RCOT. Moreover, this theory supports RCOT irrespective of FR/SD’s intention. Relying on the public property theory bypasses issues pertaining to the scope of consent in RCOT and supports designation of the organs as public property upon death by neurological criteria [30]. Finally, while the idea of organs as inheritance property in the case of Pattern 2 implies that the intention of FD who wishes for RCOT is respected even after their death, this is no longer an issue in the case of Pattern 4; neither the intention of FR/SD nor that of FD is relevant in this case.

### Argument 2 – RCOT is ethically impermissible

In the case of Pattern 4, RCOT is ethically impermissible from the perspective of consent.

As for Pattern 2 (FD, “Agree”; FR/SD, “Disagree”), RCOT is ethically impermissible when argued in terms of self-ownership. In other words, if FR/SD denies consent for RCOT, this intention is to be respected given that the ownership of the heart is considered to have already been transferred to FR/SD at the time of decision-making regarding RCOT.

In Pattern 3 (FD, “Disagree”; FR/SD, “Agree”), the issue is whether it is permissible to allow FD’s intention to prohibit RCOT, even though FR/SD wishes for RCOT. In this case, the assertion regarding FD’s self-ownership of the heart is respected to the maximum extent possible. This concept of self-ownership raises issues around designated organ donation (DOD), in which a donor designates a recipient. DOD is controversial, but is supported primarily because it increases donors, even if only slightly. In the case of DOD, FD provides consent to donate their heart to the designated recipient only (e.g., a relative). If the recipient (i.e., FR/SD) wants to re-donate the heart to SR, how should the initial consent of FD be treated? If DOD is permissible and FD’s ownership of the heart is respected to the fullest, then there may be discrepancies that emerge with regard to FR/SD’s intention. Thus, some would argue that it is ethically impermissible for FR/SD to override FD’s intention and re-transplant the heart to someone who is not in a kinship relationship with, or otherwise designated by, FD. Moreover, allowing FD’s organ to be regarded as private property gives rise to yet another ethical issue. That is, an ethical concern might arise relating to the “commercialization” of organs, or that transplant medicine perceives organs as “goods” or “commodities.”

When arguing the ethical impermissibility of RCOT, Pattern 1 (both FD and FR/SD “Agree”) presents the most difficult situation. As a challenging argument, we discuss this situation from two aspects, which include the ethics of recycle/reusing and identity issues.

Unlike ordinary transplantation, which is performed only once, or retransplantation involving the same recipient, RCOT results in the use of a donated organ (i.e., heart) in three or more human bodies. It is possible for surgeons to transplant the donated organ into the bodies of four or more people. Whether social consensus on this “organ recycle/reusing” has been established or not remains unclear. Some may perceive “recycle/reusing” negatively. If the image linked to RCOT is that of a heart that leaves the donor’s body and wanders from one recipient to the next, does this evoke a sense of disgust? If so, is this disgust something we should overcome, as it arises simply because we are unaccustomed to the concept of RCOT?

There are at least two reasons supporting the argument that organ recycling creates emotional resistance. The first reason relates to instrumentalization of donated organs. Through the continual recycling, the organ transforms from a valuable gift from the donor to the recipient, to (eventually) an instrumentalized object, or thing. As instrumentalized objects are things that can be price-tagged, the instrumentalization of organs leads directly into organ trafficking. As discussed later on, our society does not allow organ trafficking, and organ trafficking evokes emotional resistance within us. The second reason creating emotional resistance toward organ recycling is the personalization of things (or instrumentalized objects). Through its continual recycling, the history of each owner accumulates within the particular organ. In other words, the organ will inevitably contain its own unique history. This means that the organ will be shouldered with some form of inherent memory, which would form what might be called a ‘pseudo personality.’ One historical example that may help to explain this concept further is that of the Hope Diamond. While this particular gem currently resides in the Smithsonian National Museum of Natural History, it has been owned by many human beings in the past. Many of the prior owners were known to have lived tragic lives, and thus, the Hope Diamond has come to be viewed as a personalized object that brings about a curse to its owner. This same concept may be said to apply to the reuse of organs as well. Notably, as discussed later, the unique memory of the organ could potentially damage the identity of the recipient as the new owner.

Many people find no ethical problem with the expression “recycling paper,” and putting that into action might even seem ethically correct. However, some might feel disgust when they hear the expression “recycling human organs,” at least at this time. This suggests that paper and human organs cannot be lumped in the same category, and the difference is likely rooted in the notion that “organs are precious.” This notion stems from the idea that as they are part of the human body, organs should be accorded respect in a moral sense; hence, they must not be recycled.

Next, let us discuss issues related to identity. The ethical concern that the act of organ transplantation, which turns someone else’s organ into a part of one’s body, threatens the identity of the self has been discussed previously [31]. This concern is common to normal multiple cardiac retransplantation; that is, frequent exchanges of organs may make the boundary of the self unclear.

This ethical concern might grow even deeper in RCOT with the notion of organ recycle/reuse. A recipient receives an organ that has been used by multiple individuals; this pluralism might obscure the clear outline of the donor’s identity. Therefore, RCOT, which utilizes an organ that has been passed around among multiple human beings, could generate a sense that multiple nameless others exist within one’s own body. Such a sense might be somewhat unique, and likely differs from that associated with retransplantation, in which one recipient receives transplants multiple times. In this regard, there might also be a risk of impacting the formation of recipient identity—another unique aspect of RCOT.

In sum, when recycle/reuse and identity issues are taken into consideration, it is possible to argue—even in the case of Pattern 1—that RCOT is ethically impermissible.

## Discussion

### Judgment

An ethical dilemma emerges when RCOT is performed, or not performed, against the will of FD and/or FR/SD. Even if priority is given to FR/SD’s intention, we can argue that RCOT is ethically permissible, or that RCOT is ethically impermissible, both in light of discussions relating to the transfer of ownership rights. To summarize, one can argue that RCOT is ethically permissible on the basis of the gift-like nature of organs and their value as inheritance property, or based on the public property theory. On the other hand, the position that RCOT is ethically impermissible can be made on the grounds of organ recycling and identity issues.

In our view, the standpoint that RCOT is ethically permissible is more tenable, for three reasons. First, consider the case of Pattern 3 from the standpoint of RCOT being ethically impermissible: If it is ethically permissible to prioritize the intention of FD (who denies consent for RCOT) over that of FR/SD (who desires RCOT), then the FD’s intention for DOD (and similarly, one’s intention to subject their organs to commercial use) would equally be considered ethically permissible. However, as discussed above, DOD is a controversial system and the commercial use of organs is absolutely unacceptable, as supported by a large body of literature. Therefore, it is ethically impermissible to give priority to FD’s intention to deny consent for RCOT, over the intention of FR/SD who wishes to donate the transplanted organ.

Second, debates over organ recycling might simply stem from our personal, emotional responses to RCOT, as we have not yet been accustomed to the concept of RCOT. In this regard, the argument that relies on disgust toward organ recycling—by distinguishing recycling of organs from recycling of things—can be criticized as being no better than mere intuition. This is because, as Bentham stated in the “principle of sympathy and antipathy” [32], it lacks compelling ethical justification to prohibit any sort of action for reasons that originate from one person’s feeling of disgust. In other words, that this disgust is not solely based on the emotion of a single individual needs to be demonstrated, at least with some empirical data. However, even with such data, there is no guarantee that prohibition of RCOT can be ethically justified, considering the fact-value issues.

The third point is the weakness of the argument about identity. Personal identity can be classified as qualitative identity and numerical identity. We can say that two things are qualitatively identical if they share the same properties, and that they maintain numerical identity when they persist throughout a period of time. If, by any chance, a change in one’s qualitative identity is brought about by RCOT, such a change is considered an extension of personality change that we experience on a daily basis, and therefore not an important change. If our numerical identity is changed by RCOT, then that is a problem—although by adopting psychological criterion instead of physical criterion (the former regards personal numerical identity as memory continuity or memory connectedness and is considered more tenable than the latter, which requires bodily diachronic continuity, in addressing numerical identity [33]), we can say that RCOT would not threaten numerical personal identity. Therefore, judging that RCOT is ethically impermissible by appealing to issues of identity requires a counter-argument to psychologism with regard to numerical personal identity; this is not an easy task. Based on these three points, we consider the standpoint that RCOT is ethically permissible to be more tenable.

## Clinical implications

How will this ethical issue relating to consent affect consent acquisition in clinical practice? First, during the informed consent process, it is desirable that an additional explanation be provided to FD with regard to the possibility that the transplanted organ might be passed on to another recipient (e.g., SR and further).

This can be achieved by adding a consent item relating to retransplantation or RCOT on donor cards or registries. At the same time, SR should be informed of the fact that retransplantation involves a heart that has once been transplanted to another person, and an explanation of currently available evidence with regard to the risks and benefits of RCOT.

## Conclusion

To date, the number of cases in which RCOT was performed worldwide is still low. However, RCOT can be adopted as a way to address the current situation of absolute organ shortage. Although sufficient evidence regarding the medical risks of RCOT is lacking, at least the medical quality of reused organs should be examined carefully. When discussed from the legal perspective, there are no issues with RCOT in either the US or Japan. Perhaps, there are few legal problems in other countries as well. Legal aspects should be handled according to the domestic regulations of each country.

The argument based on self-ownership of organs prioritizes FR/SD’s will for RCOT, but leads to ethical concerns about DOD and the commercial use of organs. When RCOT is performed, or not performed, against the will of FD and/or FR/SD, the ethical issues are serious. Although organ recycling and identity issues may render RCOT ethically impermissible, arguments based on them are relatively weak. Thus, we conclude that RCOT is ethically permissible. The gift-like nature of organs, their value as inheritance property, and the public property theory of organs support the argument that RCOT is ethically permissible. These are not deontological concepts, but supererogatory and virtuous concepts. Thus, we can say not only that RCOT is ethically permissible, but is in fact ethically desirable. But this discussion remains open. In addressing these issues, there may be a need to change the way informed consent (including the family) is obtained prior to regular organ transplantation, as well as how explanations are provided to SR in the future. In addition to the original ethical issues inherent to RCOT, others that have been previously noted in relation to conventional cardiac transplantation or retransplantation become even deeper. The transplant community worldwide is expected to carry out wider and deeper discussions concerning RCOT as well as the reuse of other organs.

## Abbreviations

DNC: Death by neurological criteria
DOD: Designated organ donation
FD: First donor
FR: First recipient
JTON: Japan Organ Transplant Network
RCOT: Reuse Cardiac Organ Transplantation
SD: Second donor
SR: Second recipient
US: United States
